# Selenium- and/or Zinc-Enriched Egg Diet Improves Oxidative Damage and Regulates Gut Microbiota in D-Gal-Induced Aging Mice

**DOI:** 10.3390/nu16040512

**Published:** 2024-02-13

**Authors:** Qiaocui Liu, Yulin Wang, Yuan Wan, Yu Liang, Yali Tan, Mengya Wei, Tao Hou

**Affiliations:** 1 Key Laboratory of Egg Processing, Ministry of Agriculture and Rural Affairs, Wuhan 430000, China; liuqiaocui_24@webmail.hzau.edu.cn (Q.L.); wanyuan@webmail.hzau.edu.cn (Y.W.); liangyu@webmail.hzau.edu.cn (Y.L.); tanyali@webmail.hzau.edu.cn (Y.T.); weimengya@webmail.hzau.edu.cn (M.W.); 2 College of Food Science and Technology, Huazhong Agricultural University, Wuhan 430070, China; shendanfzb@163.com; 3 Key Laboratory of Environment Correlative Dietology, Ministry of Education, Huazhong Agricultural University, Wuhan 430070, China

**Keywords:** selenium- and/or zinc-enriched eggs, oxidative stress, cognitive impairment, intestinal flora

## Abstract

Eggs, with their high nutritional value, are great carriers for enriching nutrients. In this study, selenium- and/or zinc-enriched eggs (SZE) were obtained and their effects on ameliorating oxidative stress injury, alleviating cognitive impairment, and maintaining intestinal flora balance in a D-gal-induced aging mice model were investigated. As determined by the Y-maze test, SZE restored the learning and memory abilities and increased the Ach level and AChE activity of aging mice (*p* < 0.05). Meanwhile, supplementation of low-dose SZE increased antioxidant levels and decreased inflammation levels (*p* < 0.05). High-dose SZE increased anti-inflammatory levels but were less effective than low dose. Additionally, SZE maintained the intestinal flora balance and significantly increased the ratio of *Firmicutes* and *Bacteroidota*. *Blautia*, as a probiotic, was negatively correlated with pro-inflammatory factors and positively correlated with antioxidant levels (*p* < 0.05). These results suggest that SZE might improve organ damage and cognitive function by attenuating oxidative stress and inflammatory response and maintaining healthy gut flora.

## 1. Introduction

Aging is a process of irreversible structural and functional decline due to a gradual decline in the organism’s ability to cope with stress and maintain homeostasis under the combined influence of genetics and the environment [1]. It is associated with a gradual imbalance between the concentration of reactive oxygen species (ROS) in cells, such as lipid oxidation, increasing DNA oxidation products, and protein oxidation [2]. The accumulation of oxidative stress inhibits normal cellular function, leads to cellular aging and death, and induces a number of aging-related diseases, which are constant threats to the health of the elderly [3].

Currently, more and more attention and research is focusing on active substances with anti-aging effects [4]. Among them, selenium (Se) and zinc (Zn) are essential trace elements in the human body, and a large number of reports have shown that Se and Zn are closely related to senile diseases [5,6]. Se plays a physiological role in the organism, mainly in the form of selenoproteins, which can reduce the production of ROS and inflammatory responses, thus combating aging [7]. Glutathione peroxidase (GSH-Px), the most abundant selenoprotein in the body and the main metabolic form of resistance to oxidative stress, reduces free radical reactions by reducing organic hydroperoxides (ROOH) to alcohols (ROH) [8]. Selenoprotein W prevents H_2_O_2_-induced oxidative stress in vivo by reducing protein disulfide bonds in the cytoplasm, while selenoprotein H avoids superoxide production by inhibiting lipid peroxides [9]. Additionally, Zeng et al. confirmed that selenoproteins extracted from Se-enriched rice alleviated aging by enhancing the antioxidant activity of enzymes in the serum of aging mice [10], while Zn also plays an irreplaceable role in chronic diseases related to aging, mainly combating aging by inhibiting NADPH oxidase activity and reducing the production of OH^−^, O^2−^, and H_2_O_2_ [11]. Zn polysaccharides extracted from *Grifola frondosa* SH-05 alleviated aging by scavenging hydroxyl radicals and DPPH radicals and increasing antioxidant enzyme activities [12]. Xu et al. showed that the dietary addition of Zn lactate and Zn glycine chelate increased antioxidant enzyme activities and related gene expression in the intestine and improved antioxidant capacity [13]. Takeshi et al. reported that low expression of Zn transporter 3 (ZnT3) led to Zn deficiency in synaptic vesicles of the mossy fiber pathway, which accelerated brain aging in rats. However, few studies have reported about the combined anti-aging effects of Se and Zn.

Eggs are high in nutritional value, rich in protein, fatty acids, vitamins, small amounts of carbohydrates, and trace minerals, and play an important role in human diet and nutrition [14]. Importantly, eggs have many physiological functions that are beneficial to human health, such as immunomodulation, anticancer activity, antioxidant activity, anti-inflammatory activity, antihypertensive effect, and promotion of bone health [15], and the key to the performance of these functions is the presence of ovalbumin, ovoglobulin, phosphatide, and other active substances in eggs [16]. At present, the enrichment and conversion of Se and Zn to an organic state through plants and animals are innovative means to improve mineral potency, and egg organic conversion is one of the more efficient ways [17]. However, the nutritional functions and biological activities of Se- and/or Zn-enriched egg (SZE) have been less evaluated at this stage.

Therefore, the aim of this study was to firstly investigate the main chemical composition of SZE. The effects on oxidative damage, cognitive function, and the intestinal flora of SZE in D-galactose (D-gal)-induced aging mice were also studied. We hypothesized that SZE were effective in reducing oxidative stress damage caused by aging, thus lowering body inflammation levels and maintaining healthy gut flora.

## 2. Materials and Methods

### 2.1. Materials and Reagents

SZE and normal eggs (NE) were provided by Hubei Shendan Health Food Co., Ltd. (Wuhan, China), and all were produced from the same breed of chickens (the feeding environment was the same). Only the former hens were fed with 300 g yeast Se/t feed and 920 g Zn aminovite/t feed. D-galactose (D-gal, purity: 99%) was purchased from Shanghai Yuanye Bio-Technology Co., Ltd. (Shanghai, China). DL-Selenomenthionine (purity: 98%) was purchased from Beijing Bailingwei Technology Co., Ltd. (Beijing, China). Zinc sulfate heptahydrate (ZnSO_4_, purity: 99%) was purchased from Sinopharm Chemical Reagent Co., Ltd. (Shanghai, China). All other chemicals and reagents are of analytical grade or excellent grade. 

### 2.2. Pre-Treatment of Egg Powder

A total of 200 eggs were used for detection, of which 100 egg whites and yolks were artificially separated, and then the whole egg, egg white, and yolk were collected and homogenized using a high-speed disperser. After that, part of the egg liquid was immediately taken for moisture content detection. The remaining egg liquid was freeze-dried into powder by a vacuum freeze-drying machine (LGJ-10, Beijing, China), and the dried powder was stored at −20 °C for use. 

### 2.3. Detection of Basic Nutrients

According to the determination standards of moisture, ash, protein, total lipid, and phospholipids in food formulated by the People’s Republic of China, egg powder was detected by the direct drying method [18], burning method [19], Kjeldahl method [20], Soxhlet extraction method [21], and moly-blue colorimetric method [22], respectively. The total sugar content in the samples was detected by the phenol–sulfuric acid method [17]. Each sample was analyzed in triplicate.

### 2.4. ICP-MS Analysis

Quantities of 0.2 g of egg powder and 10 mL HNO_3_ were added into a polytetrafluoroethylene microwave digestion tank, which was covered and placed overnight, then the tank was digested in the microwave digestion instrument (Mars 6, CEM Corporation, Matthews, NC, USA) according to the heating program. The ablated samples were subsequently fixed with ultrapure water to 25 mL. High-purity multi-element standards and internal standards (Rh) were purchased from Agilent Technologies Inc. (Santa Clara, CA, USA) and were used to make standard curves. ICP-MS (PerkinElmer NexION350X, Waltham, MA, USA) was used to determine the mineral content in the ablated samples. The instrumental operating parameters are shown in Table 1. Each sample was analyzed in triplicate.

### 2.5. Animal Experimental Design

Seventy male Kunming mice (30 ± 2 g; 8 weeks old) were purchased from the Experimental Animal Center of Huazhong Agricultural University (Animal license number: SYXK2020-0084). We chose the ages of mice based on a previous study [23]. All of the mice were maintained under specific pathogen-free (SPF) conditions at a constant temperature (22 ± 2 °C) and relative humidity (55 ± 5%) in a 12/12 h light/dark cycle. The experimental procedures and animal welfare were carried out following the criteria outlined in the Guide for the Care and Use of Laboratory Animals (Eighth Edition) [24] and the related ethical regulations of the Huazhong Agricultural University. All experimental protocols were approved by the Animal Ethics Committee of the Huazhong Agricultural University (Approval Number: HZAUMO-2023-0219, Approval Date: 2023.09.04).

**Figure 1:**
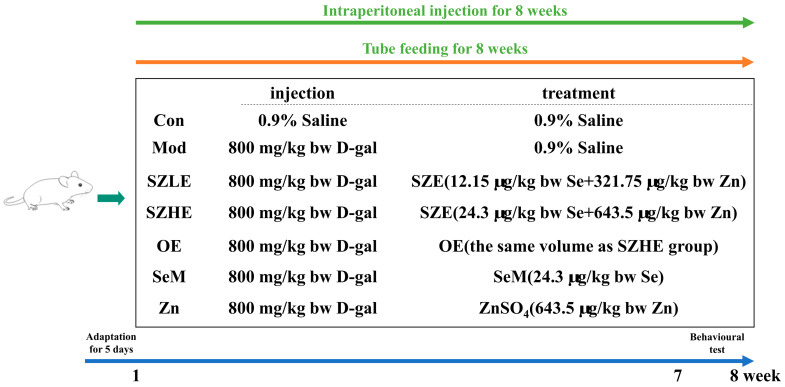
Schematic diagram of animal experiment design.

After 5 days of acclimation, each group was treated by tube feeding and the mice were randomly divided into seven groups (*n* = 10): the control group with 0.9% saline (Con), the model group with 0.9% saline (Mod), the low-dose SZE group with 12.15 µg/kg bw Se and 321.75 µg/kg bw Zn (SZLE), the high-dose SZE group with 24.3 µg/kg bw Se and 643.5 µg/kg bw Zn (SZHE), the ordinary egg group giving the same quality egg powder as SZHE (OE), the DL-Selenomenthionine group with 24.3 µg/kg bw Se (SeM), and the ZnSO_4_ group with 643.5 µg/kg bw Zn (ZnSO_4_). The Con group was intraperitoneally injected with 0.9% saline and the other groups were injected with D-gal (800 mg/kg bw) once daily for 8 weeks. Based on previous study and pre-experiment, we chose this dose of D-gal [25]. The details of the grouping and respective treatments are presented in Figure 1. The mice had free access to standard laboratory mice chow and water, and all the chemicals and eggs were administered every morning. Whereafter, behavioral analysis was performed at 1 p.m. from day 48 to day 56. On the 57th day of the experiment, the mice were fasted for 12 h, anesthetized with anhydrous ether (Sinopharm Chemical Reagents Co., Ltd., Shanghai, China) for 1 min, and euthanized by cervical dislocation after blood collection from the orbital plexus. Immediately thereafter, the organs (liver, brain, and duodenum) and cecum contents of the mice were carefully separated. Blocks of approximately 10 × 10 × 2 mm of the largest lobe of the liver and transverse halves of the brain tissue were cut out and fixed in 4% paraformaldehyde (Beijing Lanjieke Technology Co., Ltd., Beijing, China) for more than 24 h for histological analysis. The remaining brain and liver tissues, duodenum, and cecum contents were also collected in sterile EP tubes and immediately stored at −80 °C for subsequent analysis.

### 2.6. Animal’s Weight Change and Relative Organ Weight

The body weight of mice was recorded every week. The brain, liver, spleen, and kidneys of each group of mice were washed with PBS buffer, dried with filter paper, and weighed to calculate the organ index. Organ index was defined as the ratio of organ weight to body weight.

### 2.7. Spontaneous Alternating Behavior Test

The Y-maze with a wall consists of three identical arms (40 × 16 × 10 cm) with an angle of 120°. These three arms are randomly set as the left arm (the new arm), the starting arm, and the right arm (the other arm). At the same time, the VisuTrack animal behavior analysis software of Shanghai Xinrun Information Technology Co., Ltd. (version 2.0, Shanghai, China) is used to make the corresponding mark. The camera is placed above the maze to ensure that each arm is within the video range.

Before all experiments, the mice were used to adapting to the Y-maze for 2 days, during which they were free to visit all arms for 10 min. After the beginning of the experiment, the mice were put into the starting arm of the Y-maze and were free to move between the three arms for 5 min (day 48). The VisuTrack animal behavior analysis software was used to record and analyze the order of mice entering and leaving each arm within 5 min, so as to enter a batch of three consecutive entries in all three arms as the correct spontaneous alternation. The number of correct spontaneous alternations of each mouse and the total number of entries into the arm were recorded, and the correct rate of each mouse was calculated. After the test of each mouse, the excrement was cleaned and the Y-maze was wiped with 75% medical alcohol until the alcohol was completely volatilized; then, the next mouse was tested to prevent the odor from affecting the spontaneous alternating behavior of the subsequent mice.

### 2.8. New Arm Behavioral Test

Twenty-four hours after the end of the spontaneous alternating behavior test, the mice entered the new arm test stage. The new arm was blocked 6 days before the start of the formal experiment, and then the mice were allowed to move freely in the remaining arms of the Y-maze for 10 min every day. After the above process was completed, the formal test stage was entered (day 56). Firstly, for the training part, the new arm was closed with a clapboard, and the mice were placed in the other two arms and were free to move for 8 min. The mice were trained in turn, and the second part of the experiment was performed 1 h after the end of the training part. Then followed the detection part, where the baffle was removed, and the mice were randomly placed in the arm and were free to move for 5 min. The dwell time of each mouse in each arm and the number of entries in each arm between the arms within 5 min were recorded. The S value, which was the percentage of the number of times a mouse entered the new arm, was calculated and the dwell time of the mouse in the new arm was recorded. 

### 2.9. Determination of Se and Zn Content in Tissue

The liver, brain, and duodenum were freeze-dried by a freeze-drying machine (Beijing Songyuan Huaxing Technology Development Co., Ltd., Beijing, China). Then, 0.2–0.5 g samples and 10 mL concentrated nitric acid were taken in the digestion tank, and microwave digestion was carried out according to the heating program. Subsequently, the ablated sample was fixed with ultrapure water to 25 mL. High-purity Se and Zn standards were purchased at Inorganic Ventures (Lakewood, NJ, USA) and used to make standard curves. According to the determination standards of Se and Zn in food formulated by the People’s Republic of China, the contents of Se and Zn in tissue were detected by the SA-50 system (SA-50, Titan Instruments Company, Beijing, China) and the AA-6300C system (AA-6300C, Hitachi Limited, Tokyo, Japan), respectively [26,27].

### 2.10. Analysis of Ach and AChE Levels in the Brain

Brain tissue samples were added with normal saline at a mass ratio of 1:9 and mechanically homogenized under an ice bath. After centrifugation (2500 r/min, 4 °C, 10 min), the supernatant was used for the experiment. According to the manufacturer’s instructions, the concentrations of acetylcholine (Ach), acetylcholinesterase (AChE), and protein in brain tissue were detected by the microplate method and bicinchoninic acid (BCA) method, respectively. An Ach assay kit and AChE assay kit were purchased from Jiancheng Institute of Biotechnology (Nanjing, China) and BCA kits were purchased from Beijing Lanjieke Technology Co., Ltd. (Beijing, China). 

### 2.11. Analysis of Serum Antioxidant Activity

After anesthesia, orbital blood was taken from the mice, followed by low-temperature, high-speed centrifugation (3000 r/min, 4 °C, 15 min), rapid packaging, and storage at −80 °C for the experiment. The serum samples were added with normal saline with a volume ratio of 1:9, and quickly vortexed and shaken. Serum levels of malondialdehyde (MDA), total superoxide dismutase (SOD), and glutathione peroxidase (GSH-Px) were measured according to the manufacturer’s instructions. Test kits, including an MDA assay kit, SOD assay kit, and GSH-Px assay kit, were purchased from Jiancheng Institute of Biotechnology (Nanjing, China). The experimental steps were carried out as described in the kits.

### 2.12. Analysis of Oxidative Stress Injury in Liver Issue

As previously mentioned, serum samples were taken to detect alanine transaminase (ALT) and aspartate amino transferase (AST), and they were purchased from Nanjing Jiancheng Bioengineering Institute Co., Ltd. (Nanjing, China). After that, the liver tissue samples of mice were taken, added with PBS at a mass ratio of 1:9, and mechanically homogenized under an ice bath. The homogenate was then centrifuged in a low-temperature, high-speed centrifuge (5000× *g*, 4 °C, 10 min). The supernatant was quickly subpackaged and used as a 10% liver tissue homogeneous sample for the inflammatory factor detection experiment and was stored at −20 °C for testing. ELISA kits, purchased from Shanghai Huding Biotechnology Co., Ltd. (Shanghai, China), were used to detect Interleukin (IL)-6, IL-1β, and tumor necrosis factor α (TNF-α). Protein concentration was measured using a BCA kit purchased from Beijing Lanjieke Technology Co., Ltd. (Beijing, China), and all operations were performed according to the manufacturer’s instructions.

### 2.13. Gut Microbiota Analysis

Mouse cecum contents were collected and stored at −80 °C. Genomic DNA was extracted from the contents using the TIANamp Stool DNA Kit (Tiangen Biotech Co., Ltd., Beijing, China) and determined by 1.8% agarose gel electrophoresis, and the V3–V4 region of bacterial 16S rRNA was amplified using universal primers (F: ACTCCTACGGGAGGCAGCA, R: GGACTACHVGGGTWTCTAAT). Amplicons were sequenced using the Illumina novaseq6000 platform (Illumina, San Diego, CA, USA) and the results were analyzed by BioMaker (Beijing, China). 

### 2.14. Histopathological Analysis of Brain and Liver

The brain and liver samples were fixed in 4% paraformaldehyde, embedded in paraffin, cut into 5 μm sections, stained with hematoxylin–eosin (H&E), and observed under an optical microscope (Ti-Sti-S, Nikon Corporation, Tokyo, Japan). Images of brain and liver tissues were captured at a magnification of 300×.

### 2.15. Pathological Scores

Pathological scores of the liver were based on previous studies [28] and microscopic liver injury was scored by a semiquantitative scoring system: 0 indicated no significant injury and 4 indicated the presence of severe injury. These scores were assigned based on the assessment of the following histological features: cellular edema, interstitial edema, neutrophil infiltration, capillary congestion, and structural deformation. For each section, the average score of at least three observations was considered as the pathology score.

### 2.16. Statistical Analysis

All reported data are presented as the mean ± SEM. In the detection of the basic components of eggs, moisture is expressed as the wet weight (WW) of g/100 g eggs and other components are expressed as the dry weight (DW) of eggs. The content of Se and Zn in tissue was also expressed by dry weight (DW). Hypothesis testing methods included one-way ANOVA with a Duncan’s multiple range test using SPSS software (version 26.0, Chicago, IL, USA); *p* < 0.05 was considered statistically significant. The statistical diagram was formed by Origin 2021 (OriginLab Corporation, Northampton, MA, USA).

## 3. Results

### 3.1. The Basic Nutritional Components and Mineral Composition of Eggs

As shown in Table 2, there was no difference in moisture, total lipid, and ash content between SZE and NE. The protein content in a whole egg of SZE was significantly higher than that of NE (*p* < 0.05), and the total sugar content in the albumen of SZE was nearly 1.5 times higher than that of NE, which was similar to a previous study [17]. Interestingly, we found that compared with NE, the phospholipid in SZE increased significantly (*p* < 0.05), while the total cholesterol decreased significantly (*p* < 0.05), regardless of whether it was whole egg or egg yolk. 

In this study, the contents of Se and Zn in the whole egg, albumen, and yolk of SZE were significantly higher than those of NE (*p* < 0.05). Previous studies also indicated that after adding Se-enriched yeast [29]/Zn-enriched yeast [30] to the diet of hens, the content of Se/Zn in eggs increased significantly in an obviously dose-dependent manner. At the same time, we also found that the main enrichment site of Se was albumen, while Zn was mainly enriched in yolk. The analysis of heavy and toxic metals’ elements in eggs showed that the contents of As, Cr, Cd, Pb, Ni, Al (*p* < 0.05), Ag, and Cu in whole eggs of SZE were lower than NE. As an essential trace element for the human body, the contents of Fe, Mn (*p* < 0.05), and Mo increased in whole eggs of SZE. Compared with the albumen of NE, the content of Pb decreased significantly, and the content of Mg increased significantly in SZE (*p* < 0.05). The content of Ag and Cr in the yolk of SZE was also decreased significantly (*p* < 0.05).

### 3.2. SZE Regulated the Physical Condition of Mice

**Figure 2:**
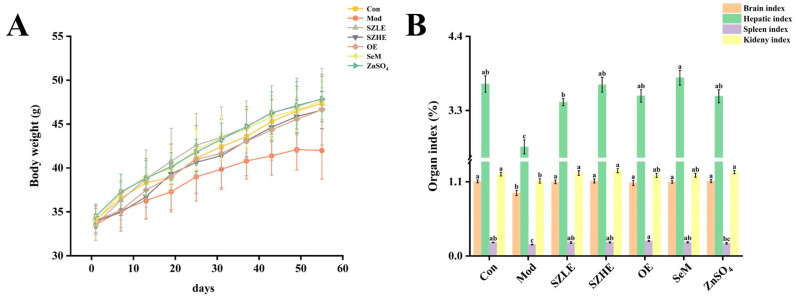
Analysis of physical condition of mice. (**A**) Body weight change. (**B**) Organ index for each group of mice. Data are presented as the mean ± SEM (*n* = 6 for each group). Different letters mean statistically significant differences at the level of *p* < 0.05.

As shown in Figure 2A, the body weight of the mice all presented an increasing trend. After 5 weeks, we observed that, compared to the Con group, the Mod group gradually showed a slower increase and dull coat luster accompanied by shedding and slower weight gain, with a tendency to decrease at a later stage, and a preference for clustering. After treatment with SZLE, SZHE, OE, SeM, and ZnSO_4_, the adverse conditions caused by D-gal induction were improved to varying degrees. 

After 8 weeks of D-gal injection, the organ index of the Mod group was significantly reduced compared with the Con group (*p* < 0.05, Figure 2B), indicating that D-gal-induced atrophy in the organs of mice; however, the possibility of the reduction of the organ index was attenuated after the intervention of SZE, OE, SeM, and ZnSO_4_, and the organ indexes of the SZLE and SZHE groups were able to return to normal levels.

### 3.3. Distribution of Se and Zn in Tissue

**Figure 3:**
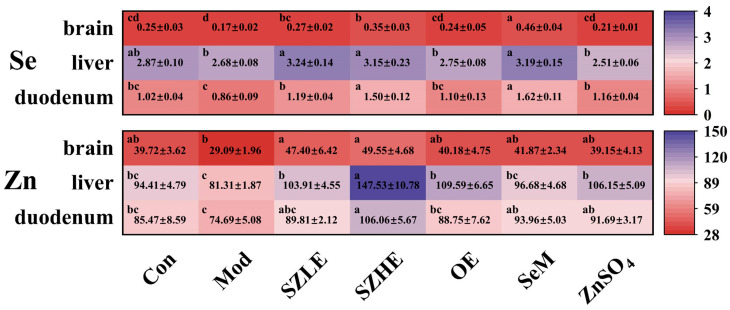
Distribution of Se and Zn in tissue (mg/kg DW). Data are presented as the mean ± SEM (*n* = 6 for each group). Different letters mean statistically significant differences at the level of *p* < 0.05.

As shown in Figure 3, Se and Zn were mainly enriched in the liver, followed by the duodenum and the brain. There was no significant difference in the Se content of each organ between the SZHE group and the SZLE group (*p* < 0.05). The content of Zn in each organ of the SZHE group was higher than the SZLE group, especially in the liver, where the SZHE group was significantly higher than the SZLE group (*p* < 0.05). At the same time, the contents of Se and Zn in the organs of the SZLE and SZHE groups were higher than the Con and Mod groups. The content of Se in the brain and duodenum of the SZHE group was significantly higher than the OE and ZnSO_4_ groups (*p* < 0.05). Additionally, the content of Zn in the liver of the SZHE group was significantly higher than the OE, SeM, and ZnSO_4_ groups (*p* < 0.05).

### 3.4. SZE Alleviated D-Gal-Induced Oxidative Stress

**Figure 4:**
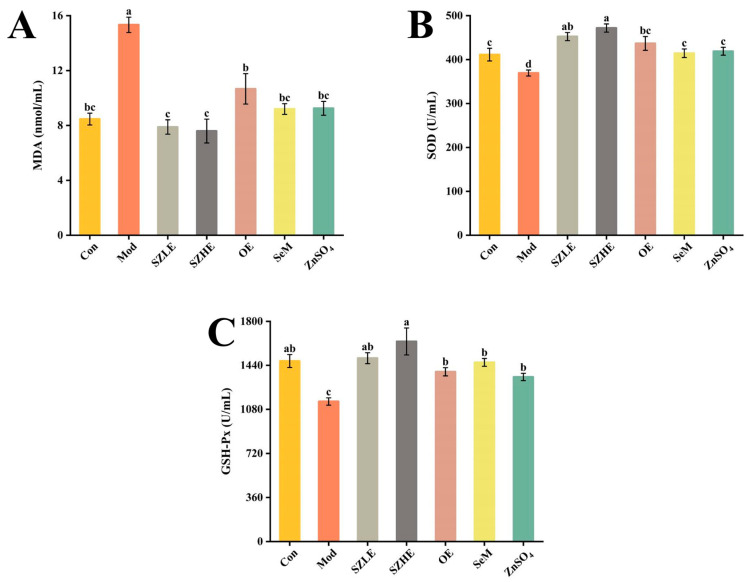
Effects of SZE on antioxidant level in serum of mice. (**A**) MDA in serum of mice. (**B**) SOD in serum of mice. (**C**) GSH-Px in serum of mice. Data are presented as the mean ± SEM (*n* = 6 for each group). Different letters mean statistically significant differences at the level of *p* < 0.05.

Compared with the Con group, the Mod group showed a significant decrease (*p* < 0.05) of SOD and GSH-Px and a significant increase (*p* < 0.05) of MDA levels after 8 consecutive weeks of subcutaneous injections of D-gal (Figure 4A–C). SZE supplementation obviously increased the levels of SOD and GSH-Px and showed declined MDA content (*p* < 0.05). SOD, GSH-Px, and MDA in the OE, SeM, and ZnSO_4_ groups were all significantly different from Mod (*p* < 0.05) and were able to recover to Con levels, but not as well as SZLE and SZHE (*p* < 0.05).

### 3.5. SZE Reversed Learning Memory Impairment Caused by D-Gal

**Figure 5:**
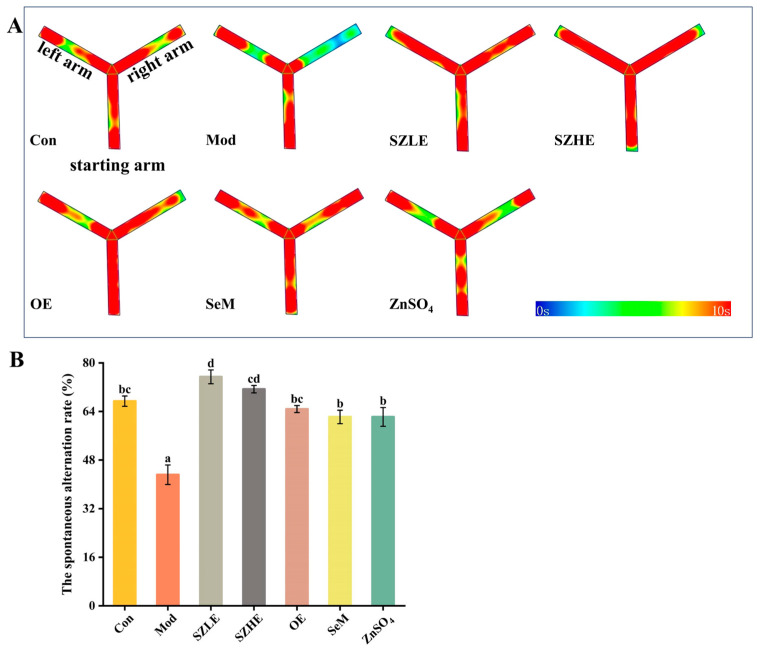
Effects of SZE on spontaneous alternating behavior in mice. (**A**) Thermograms of spontaneous alternating behavior experiments. (**B**) Spontaneous alternation rate of mice in the Y-maze. Data are presented as the mean ± SEM (*n* = 6 for each group). Different letters mean statistically significant differences at the level of *p* < 0.05.

Spontaneous alternation behavioral experiments are commonly used to evaluate the short-term learning memory ability of mice, in which a higher rate of correct spontaneous alternation represents a stronger short-term working memory ability. It can be visualized that the mice in the Mod group preferred to move in the left and starting arms of the Y-maze, while the Con group and the rest of the treatment groups moved more evenly in all three arms (Figure 5A). By further analyzing the spontaneous alternation rate, we found that the alternation rate in the Mod group reduced to approximately 40%, which was significantly lower than in the Con group (*p* < 0.05, Figure 5B). This indicated that the short-term learning memory of the mice was significantly impaired, while the alternation rate could be restored to the level of the Con group after the interventions of SZE, OE, SeM, and ZnSO_4_, and the alternation rate of SZLE was significantly higher than the Con group (*p* < 0.05), which could be up to nearly 80%.

**Figure 6:**
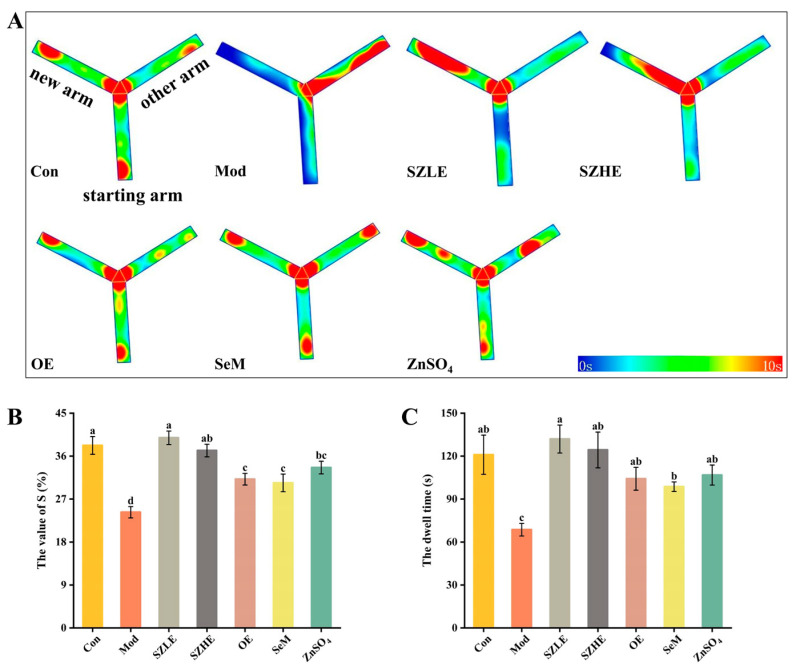
Effects of SZE on the new arm test in mice. (**A**) Thermograms of the new arm experiment. (**B**) S value for mice entering the new arm. (**C**) Dwell time of mice in the new arm. Data are presented as the mean ± SEM (*n* = 6 for each group). Different letters mean statistically significant differences at the level of *p* < 0.05.

The mice in the Mod group preferred to stay in the other arm (the non-new arm), while the SZLE/SZHE group, in contrast, preferred to explore the new arm (Figure 6A). From Figure 6B,C, the S value and dwell time of the Mod group were significantly lower than the Con group. The Mod group only stayed in the new arm for approximately 60 s, which was about half the time of the Con group, indicating impaired spatial learning and memory in the mice treated with D-gal. The S value of all administered groups was significantly higher than the Mod group (*p* < 0.05), while only the SZLE and SZHE groups were able to return to normal levels, with both exceeding 36%. The SZLE group had the longest dwell time in the new arm (more than 120 s) of all the treatment groups followed by the SZHE, and the rest of the treatment groups’ dwell times were all significantly longer than the Mod group (*p* < 0.05).

### 3.6. SZE Improved D-Gal-Induced Brain Dysfunction

**Figure 7:**
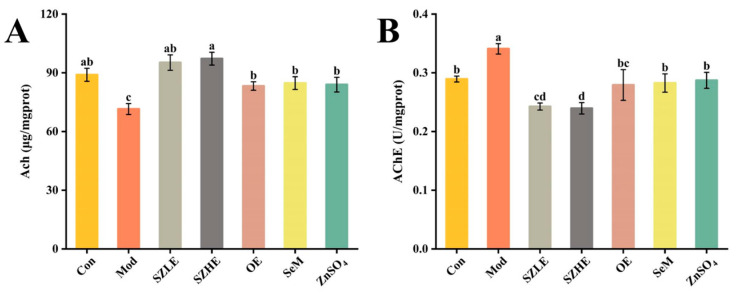
SZE significantly ameliorates cognitive dysfunction in D-gal-induced aging mice. (**A**) Ach levels in mice brain. (**B**) AChE levels in mice brain. Data are presented as the mean ± SEM (*n* = 6 for each group). Different letters mean statistically significant differences at the level of *p* < 0.05.

In Figure 7A,B, Ach levels were significantly reduced and AChE viability was significantly elevated in the Mod group compared to the Con group (*p* < 0.05), suggesting that the cognitive functions of the mice were severely impaired. Compared with the Mod group, the Ach level in the SZLE/SZHE group was significantly higher (*p* < 0.05) and was able to recover to the normal level (*p* < 0.05); the AChE enzyme activity was significantly lower compared with that in the Con group. The mice in the OE, SeM, and ZnSO_4_ groups had a significantly higher Ach level and lower AChE enzyme activity compared with the Mod group (*p* < 0.05), and all of them were able to restore the normal cognitive function of the aging mice. More importantly, the recovery effect of the SZLE/SZHE group was significantly better than the other three groups (*p* < 0.05).

**Figure 8:**
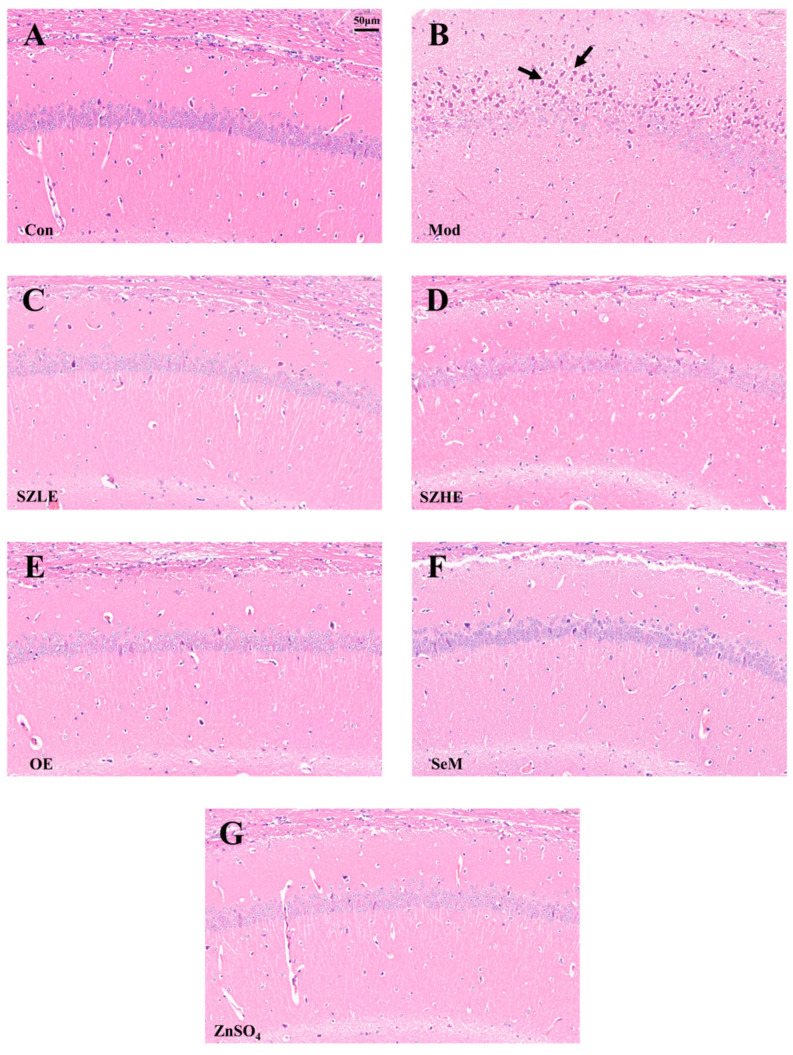
SZE significantly ameliorates cognitive dysfunction in D-gal-induced aging mice. (**A**–**G**) H&E-stained images of brain tissue from each group. The arrows in the figure mean to highlight neurons’ changes in the CA1 region. Data are presented as the mean ± SEM (*n* = 6 for each group).

Pathohistological changes in the CA1 region of the cerebral hippocampus are shown in Figure 8A–G. The neurons in the CA1 area of the Con group were elliptical or rounded, with a large number of neurons tightly and neatly arranged and clearly layered, whereas in the Mod group, a large number of neurons in the CA1 region were seen to be fixed and deeply stained, with poorly demarcated cytoplasmic nuclei and enhanced basophilicity, as well as being sparsely arranged and untidy. Compared with the Con group, the neurons in the CA1 area of the SZLE/SZHE group had no obvious changes and were neatly arranged; meanwhile, the neurons in the CA1 area of the rest of the intervention groups also had no obvious lesions.

### 3.7. SZE Attenuated D-Gal-Induced Hepatic Impairment and Inflammation

**Figure 9:**
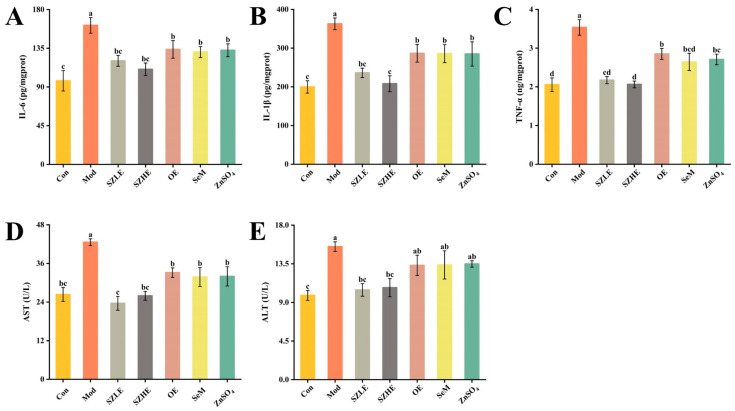
SZE significantly ameliorates D-gal-induced hepatic impairment and reduces inflammation levels. (**A**) IL-6 in liver of mice. (**B**) IL-1β in liver of mice. (**C**) TNF-α in liver of mice. (**D**) AST in serum. (**E**) ALT in serum. Data are presented as the mean ± SEM (*n* = 6 for each group). Different letters mean statistically significant differences at the level of *p* < 0.05.

D-gal induction resulted in significantly higher (*p* < 0.05) levels of IL-6, IL-1β, and TNF-α for the Mod group compared to the Con group (Figure 9A–C), whereas SZLE/SZHE treatments showed significantly lower (*p* < 0.05) levels of IL-6, IL-1β, and TNF-α compared to Mod and were able to recover to Con levels. However, in the OE, SeM and ZnSO_4_ treated groups, although the levels of IL-6, IL-1β, and TNF-α did not reach the Con level, they recovered significantly compared to the Mod group.

As shown in Figure 9D,E, after continuous injection of D-gal for 8 weeks, the serum ALT and AST levels of the Mod group were significantly higher than the Con group. The ALT and AST levels of the SZLE/SZHE group were significantly lower than the Mod group (*p* < 0.05) and recovered to the level of the Con group. Only AST was significantly lower than the Mod group in the remaining treatment groups (*p* < 0.05), while ALT did not differ significantly from the Mod group.

**Figure 10:**
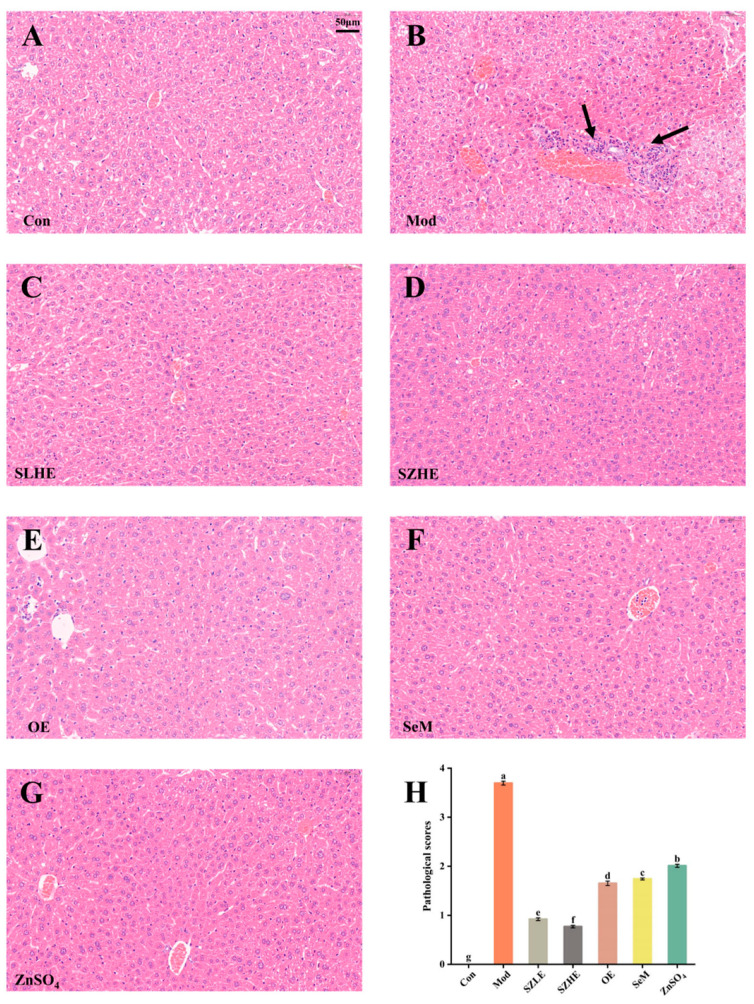
Histological analysis of mice liver. (**A**–**G**) H&E-stained images of liver tissue from each group. The arrows in the figure mean to highlight the liver inflammatory lesions. (**H**) Pathological scores of the liver. Data are presented as the mean ± SEM (*n* = 6 for each group). Different letters mean statistically significant differences at the level of *p* < 0.05.

The pathological features of hepatocyte senescence refer to the increase in the size of the nucleus and the deep staining of the nucleoplasm [31]. In the Con group, hepatocytes were regular in shape, with well-arranged cords, and vacuolar degeneration, inflammatory cell infiltration, and necrosis were not observed. As shown in Figure 10A–G, compared with the Con group, the Mod group showed enlarged hepatic interstitial spaces and deep nuclear staining, and a large number of inflammatory cell infiltration and necrosis were observed (black arrows). No necrotic areas were seen in the SZLE/SZHE group, and the phenomenon of inflammatory cell infiltration was markedly reduced with varying degrees of improvement. Additionally, inflammatory cell infiltration in the livers of the rest of the treatment groups was also reduced. The results of pathological scores showed that the Mod group was significantly higher than the Con group. Pathological scores after SZE supplementation were significantly lower than the Mod group and significantly lower than the rest of the intervention groups (*p* < 0.05, Figure 10H). 

### 3.8. Effects of SZE on Intestinal Flora in Aging Mice

**Figure 11:**
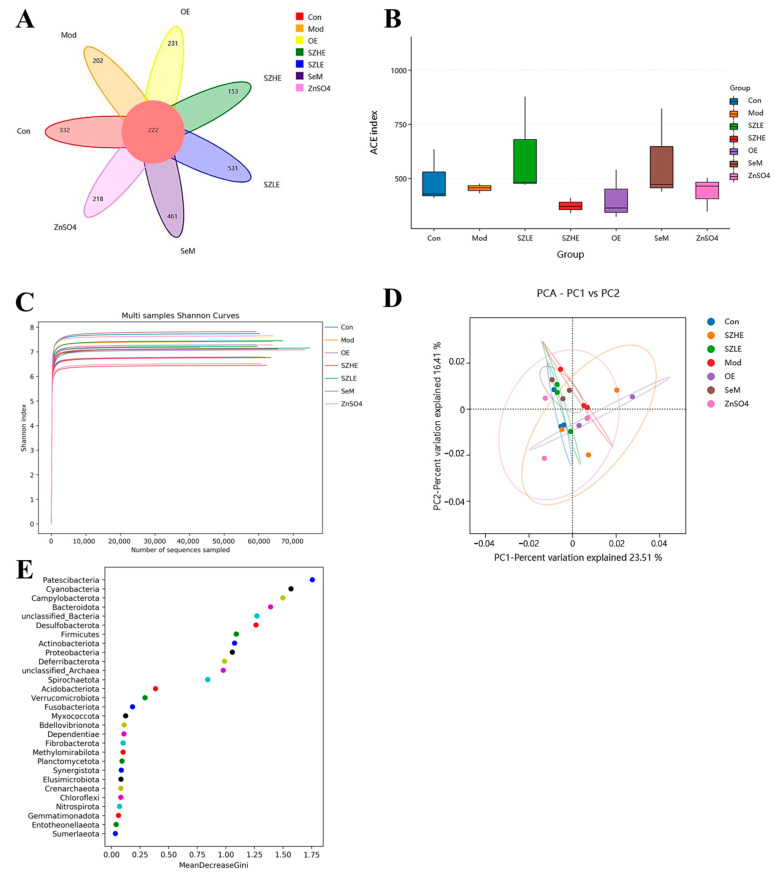
SZE significantly regulates the gut microbiota. (**A**) Venn diagram of different groups. (**B**) Student’s t-test for the ACE index. (**C**) Shannon index curve of α diversity. (**D**) Analysis of β diversity among different groups by PCA. (**E**) Phylum level random forest analysis. Data are presented as the mean ± SEM (*n* = 6 for each group).

In this study, the composition and abundance of gut microorganisms were analyzed by 16 rRNA gene sequencing of bacteria in the contents of the mouse cecum, as shown in Figure 11. In the Venn diagram, there were a total of 222 OUTs in each group, with the number of species unique to the SZLE group being the highest among all intervention groups (Figure 11A). Microbial α diversity, as reflected by the ACE index, indicated that although all medication groups had an effect on the α diversity of the intestinal flora, the effect was not significant, with the ACE index of the SZLE group being slightly higher than the Mod group (Figure 11B). The Shannon index curve flattened out, indicating that the amount of sequencing data in this study was large enough to reflect the vast majority of microbial information in each group (Figure 11C). PCA was used to analyze the β diversity between the groups. The results showed that the flora compositions of the Con and Mod groups were completely different, while the flora composition of mice supplemented with low-dose SZE was close to that of the Con group. Compared with the Con group, the flora composition of the SZHE, OE, SeM, and ZnSO_4_ groups were very different from that of the Mod group (Figure 11D). Random forest analysis at the phylum level showed that the top five with important effects on the differences between the groups were *Patescibacteria*, *Cyanobacteria*, *Campylobacterota*, *Bacteroidota*, and *unclassified _ Bacteria* (Figure 11E).

**Figure 12:**
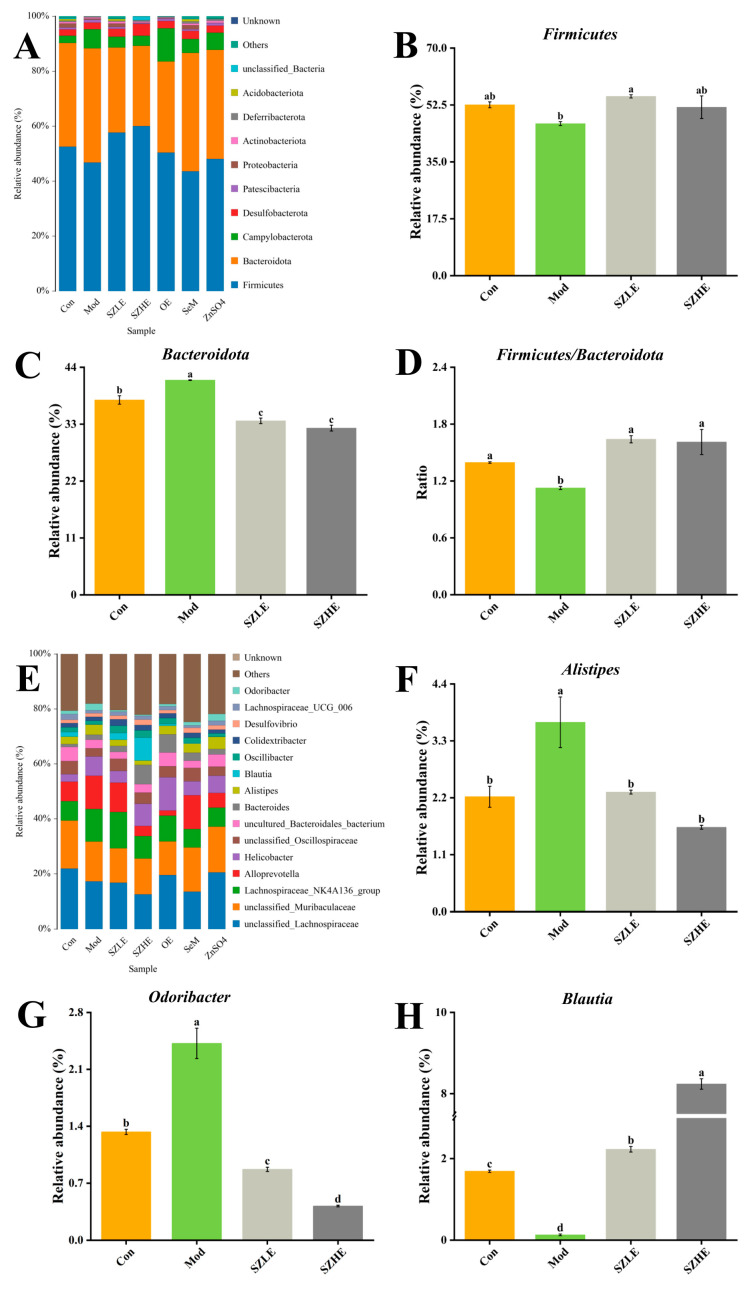
SZE significantly regulates the gut microbiota. (**A**) Intestinal microbial distribution at phylum level. (**B**) The relative abundance of *Firmicutes* at the phylum level. (**C**) The relative abundance of *Bacteroidota* at the phylum level. (**D**) The ratio of *Firmicutes* and *Bacteroidota*. (**E**) Intestinal microbial distribution at genus level. (**F**–**H**) The relative abundance of *Alistipes*, *Odoribacter*, and *Blautia* at the genus level. Data are presented as the mean ± SEM (*n* = 6 for each group). Different letters mean statistically significant differences at the level of *p* < 0.05.

Microbial community analyses at the phylum level in each group of mice showed that the major taxonomic units included *Firmicutes*, *Bacteroidota*, *Campylobacterota*, *Desulfobacteroa*, and *Patescibacteria* (Figure 12A). Compared with the Mod group, the relative abundance of *Firmicutes* was significantly higher (*p* < 0.05) in the SZLE group and the relative abundance of *Bacteroidota* was significantly lower (*p* < 0.05) in the SZLE and SZHE groups, along with a significant increase in the proportions of *Firmicutes* and *Bacteroidota* in these two groups (Figure 12B–D, *p* < 0.05). In the bar chart of the distribution of gut flora at the genus level (Figure 12E), the relative abundance of *Alistipes* and *Odoribacter* was significantly higher and *Blautia* was significantly lower in D-gal-induced aging mice, whereas the relative abundance of *Alistipes*, *Odoribacter*, and *Blautia* was significantly higher than the Mod group after administration of the SZE intervention (Figure 12F–H, *p* < 0.05). 

**Figure 13:**
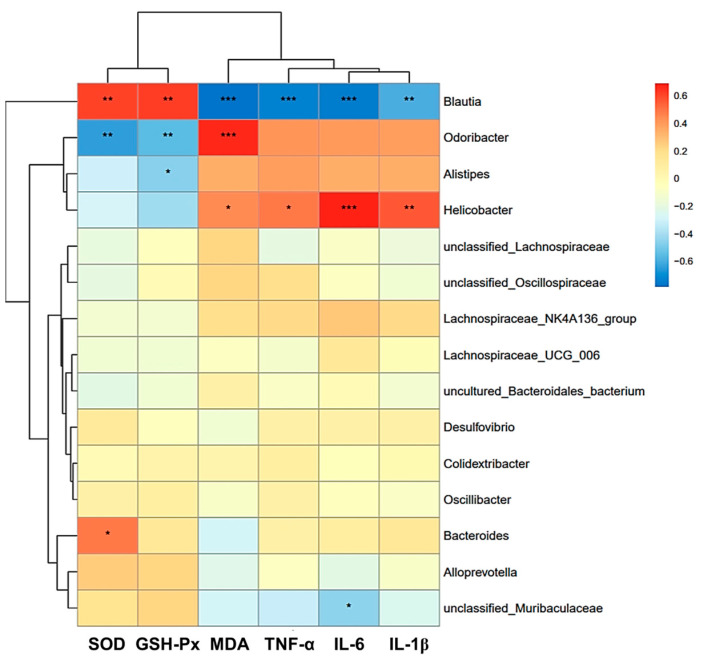
Analysis of the relationship between the relative abundance of intestinal flora and oxidative stress markers and inflammatory factors by Pearson correlation heat map of environmental factors at the genus level. Data are presented as the mean ± SEM (*n* = 6 for each group). * *p* < 0.05 versus Con group, ** *p* < 0.01 versus Con group, *** *p* < 0.001 versus Con group.

Additionally, the Pearson correlation heatmap showed potential correlations between antioxidant levels and inflammation levels with gut microflora (Figure 13). *Unclassified_Muribaculaceae* showed a significant negative correlation with IL-6, and *Bacteroides* showed a strong positive correlation with SOD (*p* < 0.05). MDA, TNF-α, IL-6, and IL-1β were positively correlated with *Helicobacter* and negatively correlated with *Blautia* (*p* < 0.05). SOD and GSH-Px were positively correlated with *Blautia* and negatively correlated with *Odoribacter* (*p* < 0.05). Meanwhile, *Odoribacter* was positively correlated with MDA (*p* < 0.05). *Alistipes* was negatively correlated with GSH-Px (*p* < 0.05).

## 4. Discussion

A link between diet, microbiota, and aging has been reported [32]. Se and Zn, as trace elements required by the human body, both have physiological functions, such as enhancing immunity, delaying aging, maintaining intestinal health and antioxidant levels, etc., and biofortification methods to achieve the supplementation of Se and Zn elements in the diet are currently innovative means. Therefore, it is important to utilize diet to modify the role played by microbiota in a variety of aging issues. In this study, we analyzed the effects of SZE on serum/liver/brain biochemical indicators, behavior, and intestinal microbes in D-gal-induced aging mice, indicating that SZE can delay aging by reducing oxidative stress, improving cognitive impairment, remodeling intestinal flora, and maintaining intestinal health.

### 4.1. SZE Can Resist Oxidative Stress Aging and Improve Cognitive Impairment

Oxidative stress is one of the major contributing factors to aging injury, and the accumulation of ROS produced by cells in the organism creates an imbalance between ROS production and clearance, and excessive ROS induces cellular damage, inflammation, and cognitive impairment [33]. Prolonged exposure to high concentrations of D-gal can cause disruption of the body’s glucose generation and contribute to the accumulation of ROS [34]. Late glycosylation end products (AGEs) produced during the metabolism of D-gal are also important contributors to neuronal damage and cognitive deficits [35]. Meanwhile, the liver plays an important role in glycolipid metabolism, and oxidative stress induced by excess D-gal could cause liver dysfunction [36]. Chronic inflammation is causally linked to the cellular redox state, and one of the changes during aging is a dysregulated immune response, which in turn leads to the development of systemic chronic inflammation. Cytokines among pro-inflammatory mediators are responsible for the development of chronic inflammation, e.g., IL-6, TNF-α, and their receptors are upregulated in senescent cells and tissues [37]. 

The results of this study showed that the composition of SZE was significantly altered, in which the content of phospholipids, Se, Zn, Fe, and Mn was significantly higher than NE. The serum SOD and GSH-Px activities of aging mice were significantly increased and the levels of MDA, AST, and ALT were significantly decreased by SZE intervention. Additionally, the inflammation (TNF-α, IL-6, IL-1β) levels in the liver of mice were reduced, and the results of H&E staining of the liver showed a significant reduction in the area of inflammatory infiltration (Figure 10A–G). The levels of Ach and AChE in brain tissue were also restored to the normal level, and neurons in the CA1 region of brain tissue were tightly aligned with a reduction in the loss of neurons (Figure 8A–G). In the Y-maze experiment, the mice supplemented with SZE showed a significant improvement in spatial learning memory ability and cognitive recovery compared with other treatment groups (Figure 5 and Figure 6). Studies have shown that SOD, a family of metalloproteins that bind to Mn, Fe, and Zn, among others, are able to scavenge intracellular ROS that may damage biomolecules such as DNA, proteins, and lipids. These enzymes prevent cells from being damaged by ROS by breaking down superoxide anion into molecular oxygen and H_2_O_2_ [38]. Se is an important component of GSH-Px, which controls cellular antioxidant levels by degrading hydroperoxides. Phospholipids promote the synthesis and regeneration of lipoproteins, repair damaged cell membranes to rejuvenate them, and also have the ability to scavenge free radicals, increase metabolism, and slow down aging [39]. Xu et al. showed that yellow catfish supplemented with Mn nanoparticles had higher SOD levels and increased mRNA expression levels of SOD1 and SOD2, and decreased mRNA expression levels of TNF-α, IL-6, and IL-1β [40]. Results from different studies have shown that Se- and/or Zn-enriched probiotic diets increase the total antioxidant capacity of canine blood [41], and Se- and/or Zn-enriched *spirulina* ameliorated the deleterious effects of heat stress in summer by increasing antioxidant enzyme activity in rabbits [42]. 

The liver and duodenum are the site of Se and Zn enrichment and absorption, respectively [43]. Co-administration of Se and Zn prevented liver injury in rats [44] and reduced the level of intestinal inflammation [45]. Brain nerve cells contain a large amount of lecithin, which can be converted to release choline, which combines with acetyl-CoA to generate Ach, so lecithin can increase the activation of brain cells and improve memory [46]. At the same time, egg yolk lecithin could inhibit the activity of AChE, reduce the concentration of oxidation products, and exert neuroprotective effects. Consumption of egg yolk lecithin could improve memory and cognitive function and delay the occurrence of neurodegenerative diseases [47]. Meanwhile, Bao et al. found that egg phospholipids significantly reversed scopolamine damage in the mouse brain and exerted neuroprotective effects by inhibiting oxidative stress, improving learning memory function in mice [48]. Several recent studies have shown that chronic exposure to a low Se environment causes cognitive decline in the elderly [49], and that a reasonable dietary intake of Se, Zn, and Cu enhances cognitive function [50], and that a sensible Zn dietary formulation improves short-term memory in Zn-restricted-diet rats [51]. These results suggest that SZE may improve cognitive impairment by enhancing antioxidant capacity and suppressing oxidative stress.

### 4.2. SZE Can Maintain Intestinal Microbial Diversity and Protect Intestinal Health

As we age, the composition of the gut flora changes significantly in older adults compared to younger ones, and imbalances in the gut flora are often accompanied by decreased gut barrier function, reduced immunity, and increased risk of Alzheimer’s disease (AD), diabetes, and other diseases [52]. Impairment of the functional barrier of the intestinal tract will lead to the entry of certain microorganisms into the circulation, which will then reach the liver and cause liver dysfunction, further affecting aging through the liver–gut axis [53]. At the same time, dysbiosis of the intestinal flora will lead to low-grade chronic inflammation, oxidative stress, and the production of various toxic metabolites; alterations in the composition and function of the intestinal flora are also associated with different liver diseases during aging [54]. Gut microbiota can promote gut health by regulating the metabolism and transport of trace elements (Se, Zn, Mn, Fe, etc.) and modulating the intestinal microenvironment through absorption from food sources [55]. Wang et al. found that Se deficiency disrupted microbiota homeostasis, decreased the expression of intestinal tight junction factors, and increased intestinal permeability, and that an imbalance in the intestinal microbiota induced inflammatory liver disease injury through the TLR4 signaling pathway [56]. 

The results of the present study showed that aging mice treated with SZE had altered gut microbial diversity. PCA analysis of SZE-supplemented mice was closer to normal levels compared to the other groups. The abundance of *Firmicutes* and *Blautia* increased and the abundance of *Alistipes* and *Odoribacter* decreased (Figure 11). In correlation analysis, *Blautia* showed a significantly negative correlation with pro-inflammatory factors (TNF-α, IL-6, IL-1β) in liver and significantly positive correlation with antioxidant enzymes (SOD and GSH-Px) in serum (Figure 12). The intestinal flora of the elderly showed an increase in the abundance of *Firmicutes* and a decrease in the abundance of *Bacteroidetes*, and the ratio of the two decreased significantly [57]. In elderly patients with constipation, the relative abundance of *Blautia* reduced [58], while *Blautia* was positively correlated with mild cognitive impairment [59] and negatively correlated with TNF-α, IL-6, and IL-1β [60], which was consistent with our results. Dietary components (including micronutrients) affect gastrointestinal colonization and microflora colonization. Dietary Se affects the composition and colonization of the gut microbiota, which in turn maintains gut health by influencing selenoprotein expression [61]. Dietary Zn, on the other hand, protects intestinal health by promoting intestinal mucosal integrity and enhancing proteins associated with barrier function (occludin and zonula occludens) [62]. He et al. reported that Se-enriched kiwifruit significantly increased the abundance of beneficial bacteria in the intestine by regulating the digestion and absorption of metabolic pathways to maintain the health of mice [63]. Hou et al. also noted that supplementation with high doses of Zn amino acids (120 mg/kg) enriched and regulated the balance of intestinal flora and promoted healthy growth of calves [64]. Therefore, Se and/or Zn diets can delay aging and alleviate organ damage by regulating intestinal microorganisms. These findings together indicate that the alleviation effect of SZE on aging may be achieved by regulating intestinal flora.

## 5. Conclusions

Daily dietary SZE significantly ameliorated oxidative stress damage and memory deficits, improved spatial learning and recognition, and maintained intestinal flora homeostasis in D-gal-induced aging mice. The results of this study provide theoretical value for a rational diet to delay aging. However, there are also some limitations, like the mechanistic aspects of SZE of this study need to be further considered and studied. Therefore, we hope to study the related mechanism at a later stage and provide reference for a diet to alleviate aging and related diseases and egg product development.

## Figures and Tables

**Table 1:** Instrumental parameters of ICP-MS.

Parameter Name	Parameters
Radio frequency power (W)	1500
Plasma flow rate (L/min)	15
Carrier gas flow rate (L/min)	0.8
Helium flow rate (mL/min)	4
Atomization chamber temperature (°C)	2
Nebulizer type	Concentric
Sampling depth (mm)	8
Sampling mode	Spectrum
Number of measurements per peak	3
Repetition number	3

**Table 2:** Nutrient and mineral content of eggs (*n* = 3).

Items	Whole Egg		Albumen		Yolk	
	SZE	NE	SZE	NE	SZE	NE
Nutritional components						
Moisture (g/100 g WW)	74.49 ± 0.08	73.32 ± 0.54	86.88 ± 0.02	87.22 ± 0.02	49.18 ± 1.28	48.25 ± 1.96
Total sugar (mg/g DW)	6.24 ± 0.29	4.88 ± 0.21	30.34 ± 0.93 *	19.95 ± 0.94	1.94 ± 0.06	1.60 ± 0.93
Ash (g/100 g DW)	4.27 ± 0.03	4.23 ± 0.05	5.70 ± 0.14	5.69 ± 0.33	3.82 ± 0.10	3.72 ± 0.03
Protein (g/100 g DW)	54.31 ± 0.38 *	51.31 ± 0.39	90.87 ± 0.25	90.41 ± 0.93	35.84 ± 0.11	35.81 ± 0.16
Total lipid (g/100 g DW)	35.94 ± 0.20	36.23 ± 0.11	-	-	50.89 ± 0.09	51.06 ± 0.01
Total cholesterol (mmol/g DW)	0.027 ± 0.0001 *	0.031 ± 0.0001	-	-	0.036 ± 0.0001 *	0.049 ± 0.001
Phosphatide (mg/g DW)	59.51 ± 0.28 *	50.09 ± 0.46	-	-	89.01 ± 0.70 *	76.68 ± 0.89 *
Mineral composition (mg/kg DW)						
Se	2.32 ± 0.09 *	1.40 ± 0.02	4.27 ± 0.21 *	2.44 ± 0.24	1.60 ± 0.04 *	1.18 ± 0.07
Zn	58.61 ± 0.79 *	51.76 ± 0.38	3.36 ± 0.06 *	1.26 ± 0.17	80.87 ± 1.56 *	65.84 ± 0.86
Mg	499.79 ± 14.65	502.64 ± 5.09	1404.31 ± 11.94 *	1187.1 ± 6.74	308.03 ± 2.26	299.83 ± 5.1
Fe	76.53 ± 6.46 *	50.09 ± 2.8	16.77 ± 1.54	14.57 ± 1.44	58.12 ± 3.95	74.89 ± 3.09
Ca	2717.09 ± 6.01	2660.56 ± 19.85	534.21 ± 8.34	532.44 ± 13.39	3536.64 ± 64.19	3604.34 ± 37.19
Al	4.39 × 10^−3^ ± 2.59 × 10^−4^ *	7.80 × 10^−2^ ± 2.29 × 10^−3^	1.12 ± 0.23	1.33 ± 0.19	0.94 ± 0.03	1.41 ± 0.12
Co	7.75 × 10^−3^ ± 1.06 × 10^−3^	7.29 × 10^−3^ ± 5.73 × 10^−4^	3.63 × 10^−3^ ± 5.06 × 10^−5^	3.19 × 10^−3^ ± 1.13 × 10^−3^	1.05 × 10^−2^ ± 1.72 × 10^−3^	1.01 × 10^−2^ ± 5.37 × 10^−4^
As	8.00 × 10^−3^ ± 1.85 × 10 *	1.27 × 10^−2^ ± 1.41 × 10^−3^	1.54 × 10^−2^ ± 1.77 × 10^−5^	1.83 × 10^−2^ ± 8.13 × 10^−4^	3.74 × 10^−3^ ± 6.01 × 10^−4^	2.55 × 10^−3^ ± 4.61 × 10^−4^
Mn	1.55 ± 0.03 *	1.28 ± 0.02	1.45 × 10^−1^ ± 2.92 × 10^−3^ *	9.11 × 10^−2^ ± 1.81 × 10^−3^	1.67 ± 0.02 *	2.53 ± 0.06
Cu	2.17 ± 0.02	2.43 ± 0.11	0.97 ± 0.03 *	1.23 ± 0.03	3.52 ± 0.05 *	3.30 ± 0.01
V	6.12 × 10^−4^ ± 3.21 × 10^−4^ *	1.01 × 10^−2^ ± 2.48 × 10^−3^	7.65 × 10^−4^ ± 07.78 × 10^−5^ *	1.45 × 10^−2^ ± 3.34 × 10^−4^	1.45 × 10^−2^ ± 3.34 × 10^−4^	1.22 × 10^−2^ ± 1.54 × 10^−3^
Ni	4.52 × 10^−2^ ± 9.48 × 10^−3^ *	2.72 × 10^−1^ ± 6.27 × 10^−2^	7.17 × 10^−2^ ± 6.46 × 10^−3^	4.41 × 10^−2^ ± 2.06 × 10^−3^	9.24 × 10^−2^ ± 3.90 × 10^−3^ *	7.13 × 10^−2^ ± 1.14 × 10^−3^
Mo	0.40 ± 0.03	0.34 ± 0.01	1.43 × 10^−1^ ± 2.64 × 10^−3^ *	6.43 × 10^−2^ ± 4.96 × 10^−3^	2.22 × 10^−1^ ± 1.27 × 10^−3^	2.49 × 10^−1^ ± 3.73 × 10^−2^
Ag	1.46 × 10^−4^ ± 4.42 × 10^−4^	8.20 × 10^−4^ ± 1.26 × 10^−4^	5.73 × 10^−4^ ± 2.27 × 10^−5^	3.88 × 10^−4^ ± 5.49 × 10^−5^	3.55 × 10^−4^ ± 2.89 × 10^−5^ *	5.34 × 10^−4^ ± 6.53 × 10^−6^
Cd	6.71 × 10^−4^ ± 7.80 × 10^−5^ *	2.86 × 10^−3^ ± 6.28 × 10^−4^	3.13 × 10^−3^ ± 3.62 × 10^−4^	4.19 × 10^−3^ ± 6.85 × 10^−4^	2.53 × 10^−3^ ± 6.58 × 10^−4^	3.34 × 10^−3^ ± 9.97 × 10^−4^
Ba	1.29 ± 0.07	1.27 ± 0.02	3.58 × 10^−2^ ± 2.82 × 10^−3^ *	6.47 × 10^−2^ ± 8.41 × 10^−4^	1.42 ± 0.07 *	1.91 ± 0.09
Cr	3.75 × 10^−3^ ± 1.59 × 10^−3^ *	3.37 × 10^−2^ ± 2.64 × 10^−3^	4.40 × 10^−2^ ± 3.93 × 10^−3^	9.67 × 10^−2^ ± 8.79 × 10^−3^	8.16 × 10^−2^ ± 2.16 × 10^−2^ *	1.13 × 10^−1^ ± 1.94 × 10^−2^
Pb	4.14 × 10^−3^ ± 6.05 × 10^−4^ *	3.76 × 10^−2^ ± 3.31 × 10^−3^	4.44 × 10^−3^ ± 3.93 × 10^−3^ *	2.86 × 10^−2^ ± 6.26 × 10^−4^	3.00 × 10^−2^ ± 2.02 × 10^−3^	3.43 × 10^−2^ ± 1.11 × 10^−3^

* *p* < 0.05 versus NE group.

## Data Availability

The data used during the current study are available from the corresponding author. The data are not publicly available due to privacy.

## References

[1] Zhu S.Y., Jiang N., Tu J., Yang J., Zhou Y. (2017). Antioxidant and Anti-aging Activities of *Silybum Marianum* Protein Hydrolysate in Mice Treated with D-galactose. Biomed. Environ. Sci..

[2] Luo J., Mills K., le Cessie S., Noordam R., van Heemst D. (2020). Ageing, age-related diseases and oxidative stress: What to do next?. Ageing Res. Rev..

[3] Bouzid M.A., Filaire E., McCall A., Fabre C. (2015). Radical Oxygen Species, Exercise and Aging: An Update. Sports Med..

[4] Chen Q., Xu B.J., Huang W.S., Amrouche A.T., Maurizio B., Simal-Gandara J., Tundis R., Xiao J.B., Zou L., Lu B.Y. (2020). Edible flowers as functional raw materials: A review on anti-aging properties. Trends Food Sci. Technol..

[5] Andreini C., Banci L., Bertini I., Rosato A. (2006). Counting the zinc-proteins encoded in the human genome. J. Phys. Chem. Lett..

[6] Genchi G., Lauria G., Catalano A., Sinicropi M.S., Carocci A. (2023). Biological Activity of Selenium and Its Impact on Human Health. Int. J. Mol. Sci..

[7] Cai Z.L., Zhang J.Z., Li H.J. (2019). Selenium, aging and aging-related diseases. Aging Clin. Exp. Res..

[8] Bjorklund G., Shanaida M., Lysiuk R., Antonyak H., Klishch I., Shanaida V., Peana M. (2022). Selenium: An Antioxidant with a Critical Role in Anti-Aging. Molecules.

[9] Zhang S., Rocourt C., Cheng W.H. (2010). Selenoproteins and the aging brain. Mech. Ageing Dev..

[10] Zeng R., Farooq M.U., Zhang G., Tang Z.C., Zheng T.D., Su Y., Hussain S., Liang Y.K., Ye X.Y., Jia X.M. (2020). Dissecting the Potential of Selenoproteins Extracted from Selenium-Enriched Rice on Physiological, Biochemical and Anti-Ageing Effects In Vivo. Biol. Trace Elem. Res..

[11] Sun R.Z., Wang J., Feng J., Cao B. (2022). Zinc in Cognitive Impairment and Aging. Biomolecules.

[12] Zhang C., Gao Z., Hu C.L., Zhang J.J., Sun X.Y., Rong C.B., Jia L. (2017). Antioxidant, antibacterial and anti-aging activities of intracellular zinc polysaccharides from *Grifola frondosa* SH-05. Int. J. Biol. Macromol..

[13] Xu Y.C., Zheng H., Guo J.C., Tan X.Y., Zhao T., Song Y.F., Wei X.L., Luo Z. (2023). Effects of Different Dietary Zinc (Zn) Sources on Growth Performance, Zn Metabolism, and Intestinal Health of Grass Carp. Antioxidants.

[14] Lu J., Qu L., Shen M.M., Hu Y.P., Guo J., Dou T.C., Wang K.H. (2018). Comparison of dynamic change of egg selenium deposition after feeding sodium selenite or selenium-enriched yeast. Poult. Sci..

[15] Kovacs-Nolan J., Phillips M., Mine Y. (2005). Advances in the value of eggs and egg components for human health. J. Agric. Food Chem..

[16] Lesnierowski G., Stangierski J. (2018). What’s new in chicken egg research and technology for human health promotion?—A review. Trends Food Sci. Technol..

[17] Liu Q., Wang Q., He P., Zhang Y., Pan L.M., Chen Y., Wu H., Zhang M.M. (2022). Heat-induced gel properties and gastrointestinal digestive properties of egg white produced by hens fed with selenium-enriched yeast. Food Chem..

[18] National Health Commission of the People’s Republic of China (2016). National Food Safety Standard-Determination of Moisture in Food.

[19] National Health Commission of the People’s Republic of China (2016). National Food Safety Standard-Determination of Ash in Food.

[20] National Health Commission of the People’s Republic of China (2016). National Food Safety Standard-Determination of Protein in Food.

[21] National Health Commission of the People’s Republic of China (2016). National Food Safety Standard-Determination of Lipid in Food.

[22] National Health Commission of the People’s Republic of China (2008). National Food Safety Standard-Determination of Phospholipids in Grain and Oil.

[23] Kong S.Z., Li J.C., Li S.D., Liao M.N., Li C.P., Zheng P.J., Guo M.H., Tan W.X., Zheng Z.H., Hu Z. (2018). Anti-Aging Effect of Chitosan Oligosaccharide on D-Galactose-Induced Subacute Aging in Mice. Mar. Drugs.

[24] National Research Council (US) Institute for Laboratory Animal Research (1996). Guide for the Care and Use of Laboratory Animals.

[25] Zhuang Y.L., Ma Q.Y., Guo Y., Sun L.P. (2017). Protective effects of rambutan (*Nephelium lappaceum)* peel phenolics on H_2_O_2_-induced oxidative damages in HepG2 cells and D-galactose induced aging mice. Food Chem. Toxicol..

[26] National Health Commission of the People’s Republic of China (2017). National Food Safety Standard-Determination of Selenium in Food.

[27] National Health Commission of the People’s Republic of China (2017). National Food Safety Standard-Determination of Zinc in Food.

[28] Luo J.X., Zhang Y., Hu X.Y., Zhong S., Chen G., Wang Y.Y., Lin W., Yi C., Zhu H. (2015). The effects of modified sini decoction on liver injury and regeneration in acute liver failure induced by D-galactosamine in rats. J. Ethnopharmacol..

[29] Lu J., Qu L., Ma M., Li Y.F., Wang X.G., Yang Z., Wang K.H. (2020). Efficacy evaluation of selenium-enriched yeast in laying hens: Effects on performance, egg quality, organ development, and selenium deposition. Poult. Sci..

[30] Cornescu G.M., Panaite T.D., Untea A.E., Varzaru I., Saracila M., Dumitru M., Vlaicu P.A., Gavris T. (2023). Mitigation of heat stress effects on laying hens’ performances, egg quality, and some blood parameters by adding dietary zinc-enriched yeasts, parsley, and their combination. Front. Vet. Sci..

[31] Chen S.S., Wang H.L., Hu N. (2022). Long-Term Dietary *Lycium ruthenicum* Murr. Anthocyanins Intake Alleviated Oxidative Stress-Mediated Aging-Related Liver Injury and Abnormal Amino Acid Metabolism. Foods.

[32] Zhao T.T., Zhong S.Y., Xu J.C., Jiao W.J., Liu W.F., Huang L.H., Zhang Y.H., Zhang Y.S. (2022). PAYCS Alleviates Scopolamine-Induced Memory Deficits in Mice by Reducing Oxidative and Inflammatory Stress and Modulation of Gut Microbiota-Fecal Metabolites-Brain Neurotransmitter Axis. J. Agric. Food Chem..

[33] Fraser H.C., Kuan V., Johnen R., Zwierzyna M., Hingorani A.D., Beyer A., Partridge L. (2022). Biological mechanisms of aging predict age-related disease co-occurrence in patients. Aging Cell.

[34] Chen P., Chen F.C., Zhou B.H. (2018). Antioxidative, anti-inflammatory and anti-apoptotic effects of ellagic acid in liver and brain of rats treated by D-galactose. Sci. Rep..

[35] Hassan W., Noreen H., Rehman S., Kamal M.A., da Rocha J.B.T. (2022). Association of Oxidative Stress with Neurological Disorders. Curr. Neuropharmacol..

[36] Chen P., Lei J.X., Chen F.C., Zhou B.H. (2020). Ameliorative effect of urolithin A on d-gal-induced liver and kidney damage in aging mice via its antioxidative, anti-inflammatory and antiapoptotic properties. RSC Adv..

[37] Chung H.Y., Kim D.H., Lee E.K., Chung K.W., Chung S., Lee B., Seo A.Y., Chung J.H., Jung Y.S., Im E. (2019). Redefining Chronic Inflammation in Aging and Age-Related Diseases: Proposal of the Senoinflammation Concept. Aging Dis..

[38] Li F., Shi H.Q., Ying S.H., Feng M.G. (2015). Distinct contributions of one Fe- and two Cu/Zn-cofactored superoxide dismutases to antioxidation, UV tolerance and virulence of *Beauveria bassiana*. Fungal Genet. Biol..

[39] Kundakovic T., Dukic N.M., Kovacevic N. (2005). Free radical scavenging activity of *Achillea alexandri-regis* extracts. Fitoterapia.

[40] Xu J.J., Zhao T., Luo Z., Zhong C.C., Zheng H., Tan X.Y. (2023). Effects of dietary supplementation with manganese dioxide nanoparticles on growth, Mn metabolism and kidney health of yellow catfish *Pelteobagrus fulvidraco*. Aquacul. Rep..

[41] Ren Z.H., Zhao Z.P., Wang Y.G., Huang K.H. (2011). Preparation of Selenium/Zinc-Enriched Probiotics and Their Effect on Blood Selenium and Zinc Concentrations, Antioxidant Capacities, and Intestinal Microflora in Canine. Biol. Trace Elem. Res..

[42] Hassan F., Mobarez S., Mohamed M., Attia Y., Mekawy A., Mahrose K. (2021). Zinc and/or Selenium Enriched Spirulina as Antioxidants in Growing Rabbit Diets to Alleviate the Deleterious Impacts of Heat Stress during Summer Season. Animals.

[43] Cempel M., Janicka K. (2002). Distribution of nickel, zinc, and copper in rat organs after oral administration of nickel(II) chloride. Biol. Trace Elem. Res..

[44] Kesik V., Lenk M.K., Kurekci A.E., Acikel C.H., Akgul E.O., Aydin A., Erdem O., Gamsizkan M. (2008). Do zinc and selenium prevent the antioxidant, hepatic and renal system impairment caused by aspirin in rats?. Biol. Trace Elem. Res..

[45] Cai Y.Y., Wang X.L., Li C.M., Li F.F., Yan Z.X., Ma N., Sun M. (2022). Probiotics combined with zinc and selenium preparation in the treatment of child rotavirus enteritis. Am. J. Transl. Res..

[46] Zhao F., Li R.J., Liu Y., Chen H.Y. (2023). Perspectives on lecithin from egg yolk: Extraction, physicochemical properties, modification, and applications. Front. Nutr..

[47] Che H.X., Fu X.Y., Zhang L.Y., Gao X., Wen M., Du L., Xue C.H., Xu J., Wang Y.M. (2018). Neuroprotective Effects of n-3 Polyunsaturated Fatty Acid-Enriched Phosphatidylserine against Oxidative Damage in PC12 Cells. Cell. Mol. Neurobiol..

[48] Bao Z.J., Zhang P.L., Chen J., Gao J., Lin S.Y., Sun N. (2020). Egg yolk phospholipids reverse scopolamine-induced spatial memory deficits in mice by attenuating cholinergic damage. J. Func. Foods.

[49] Jiang K., Xie C.X., Li Z.R., Zeng H., Zhao Y., Shi Z.M. (2022). Selenium Intake and its Interaction with Iron Intake Are Associated with Cognitive Functions in Chinese Adults: A Longitudinal Study. Nutrients.

[50] Li S.Y., Sun W.J., Zhang D.F. (2019). Association of Zinc, Iron, Copper, and Selenium Intakes with Low Cognitive Performance in Older Adults: A Cross-Sectional Study from National Health and Nutrition Examination Survey (NHANES). J Alzheimers Dis..

[51] Keller K.A., Chu Y., Grider A., Coffield J.A. (2000). Supplementation with L-histidine during dietary zinc repletion improves short-term memory in zinc-restricted young adult male rats. J. Nutr..

[52] Shi X., Ma T., Sakandar H.A., Menghe B., Sun Z.H. (2022). Gut microbiome and aging nexus and underlying mechanism. Appl. Microbiol. Biot..

[53] Zou Y.F., Yan H., Li C.Y., Wen F., Jize X., Zhang C.W., Liu S.Q., Zhao Y.Z., Fu Y.P., Li L.X. (2023). A Pectic Polysaccharide from *Codonopsis pilosula* Alleviates Inflammatory Response and Oxidative Stress of Aging Mice via Modulating Intestinal Microbiota-Related Gut-Liver Axis. Antioxidants.

[54] Campagnoli L.I.M., Marchesi N., Vairetti M., Pascale A., Ferrigno A., Barbieri A. (2022). Age-Related NAFLD: The Use of Probiotics as a Supportive Therapeutic Intervention. Cells.

[55] Pajarillo E.A.B., Lee E., Kang D.K. (2021). Trace metals and animal health: Interplay of the gut microbiota with iron, manganese, zinc, and copper. Anim. Nutr..

[56] Wang G.D., Jiang Z.H., Song Y.W., Xing Y.T., He S.M., Boomi P. (2023). Gut microbiota contribution to selenium deficiency-induced gut-liver inflammation. Biofactors.

[57] Wang W., Liu F., Xu C., Liu Z.J., Ma J.G., Gu L.Y., Jiang Z.M., Hou J.C. (2021). *Lactobacillus plantarum* 69-2 Combined with Galacto-Oligosaccharides Alleviates D-Galactose-Induced Aging by Regulating the AMPK/SIRT1 Signaling Pathway and Gut Microbiota in Mice. J. Agric. Food Chem..

[58] Ma J.X., Sun J.Q., Bai H.J., Ma H.L., Wang K., Wang J., Yu X.F., Pan Y.R., Yao J.F. (2022). Influence of Flax Seeds on the Gut Microbiota of Elderly Patients with Constipation. J. Multidiscip. Health.

[59] Park S., Wu X. (2022). Modulation of the Gut Microbiota in Memory Impairment and Alzheimer’s Disease via the Inhibition of the Parasympathetic Nervous System. Int. J. Mol. Sci..

[60] Mao B.Y., Guo W.L., Liu X.M., Cui S.M., Zhang Q.X., Zhao J.X., Tang X., Zhang H. (2023). Potential Probiotic Properties of *Blautia producta* Against Lipopolysaccharide-Induced Acute Liver Injury. Probiotics Antimicrob..

[61] Kasaikina M.V., Kravtsova M.A., Lee B.C., Seravalli J., Peterson D.A., Walter J., Legge R., Benson A.K., Hatfield D.L., Gladyshev V.N. (2011). Dietary selenium affects host selenoproteome expression by influencing the gut microbiota. FASEB J..

[62] Calder P.C., Ortega E.F., Meydani S.N., Adkins Y., Stephensen C.B., Thompson B., Zwickey H. (2022). Nutrition, Immunosenescence, and Infectious Disease: An Overview of the Scientific Evidence on Micronutrients and on Modulation of the Gut Microbiota. Adv. Nutr..

[63] He K., Gao Q., Su J.X., Shang H., Meng X., Jiang S.Q., Liu D.H., Huang B. (2023). Gut Microbiome and Metabolomics Study of Selenium-Enriched Kiwifruit Regulating Hyperlipidemia in Mice Induced by a High-Fat Diet. J. Agric. Food Chem..

[64] Hou P.X., Li B., Wang Y., Li D., Huang X.Y., Sun W.Y., Liang X.J., Zhang E.P. (2023). The Effect of Dietary Supplementation with Zinc Amino Acids on Immunity, Antioxidant Capacity, and Gut Microbiota Composition in Calves. Animals.

